# CNATNet: a convolution-attention hybrid network for safflower classification

**DOI:** 10.3389/fpls.2025.1639269

**Published:** 2025-09-30

**Authors:** Pengwei Ma, Nan Lian, Leilei Dong, Yunchen Luo, Zheng Sun, Yuanjiao Zhu, Zefang Chen, Jie Zhou

**Affiliations:** College of Information Science and Technology, Shihezi University, Shihezi, China

**Keywords:** safflower classification, deep learning, CNN-attention hybrid, C2S2, AnC2f, DWClassify

## Abstract

Safflower (Carthamus tinctorius L.) is an important medicinal and economic crop, where efficient and accurate filament grading is essential for quality control in agricultural and pharmaceutical applications. However, current methods rely on manual inspection, which is time-consuming and difficult to scale. A coarse-to-fine grading framework is established, consisting of cluster-level classification for rapid assessment and filament-level fine-grained classification. To implement this framework, a lightweight hybrid network, CNATNet, is designed by integrating convolutional operations and attention mechanisms. The classical C2f feature extraction module is optimized into two components: C2S2, a lightweight convolutional variant with cascaded split connections, and AnC2f, an n-order local attention mechanism. A depthwise separable convolution-based head (DWClassify) is further employed to accelerate inference while maintaining accuracy. Experiments on a high-resolution safflower filament dataset indicate that CNATNet achieves 98.6% accuracy at the cluster level and 95.6% at the filament level, with an average latency of 1.9 ms per image. Compared with representative baselines such as YOLOv11m and RT-DETRv2s, CNATNet consistently yields higher accuracy with reduced latency. Moreover, deployment on the Jetson Orin Nano demonstrates real-time performance at 63 FPS under 15 W, confirming its feasibility for embedded agricultural grading in resource-constrained environments. These results suggest that CNATNet provides a task-specific lightweight solution balancing accuracy and efficiency, with strong potential for practical safflower quality classification.

## 1 Introduction

Efficient classification of safflower filaments is a key challenge in modern agricultural quality control, given their significant economic and medicinal value. Safflower (Carthamus tinctorius L.) is widely recognized for its pharmacological effects, including promoting blood circulation, anti-inflammation, and antioxidation (Pu et al., 2019). Benefiting from favorable climatic conditions, Xinjiang Province accounts for more than 75% of China’s safflower production, producing filaments with high active compound content, bright color, and intact structure (Lin et al., 2020). In practical production scenarios, filament color, texture, and integrity are critical indicators for assessing safflower quality. However, large-scale filament grading still relies on manual visual inspection, which suffers from high labor intensity, subjectivity, and limited scalability, failing to meet the demands of standardized and efficient production.

To address these limitations, researchers have explored various analytical techniques for safflower-specific applications. For instance, hyperspectral imaging combined with machine learning models has been successfully applied to monitor drought stress in safflower, enabling precise classification of plant health states (Salek et al., 2024). Similarly, computer-aided decision-making systems based on spectral reflectance data have been developed to optimize irrigation strategies and assess safflower quality, demonstrating the feasibility of intelligent agricultural management (Karadağ, 2022). In terms of product safety and quality control, machine learning-assisted surface-enhanced Raman spectroscopy (SERS) sensors have been employed for rapid detection of illegal dye additives in safflower products, facilitating highly sensitive and on-site hazardous substance analysis (Lin et al., 2024). Furthermore, a practical “indistinct” evaluation method, integrating bioactivity assays with visual character analysis, has been proposed to establish efficient and low-cost quality grading standards for safflower (Zhou et al., 2023). While these safflower-related studies have achieved notable progress, complementary research on saffron (Crocus sativus L.) provides valuable technical references. Methods such as E-nose combined with gas chromatography-mass spectrometry (GC-MS) (Sun et al., 2022) and UHPLC-HRMS/MS-based metabolomics (Ryparova Kvirencova et al., 2023) have shown effectiveness in detecting adulteration and ensuring product authenticity. However, these approaches often rely on sophisticated instrumentation and complex workflows, which limit their applicability for real-time, large-scale safflower classification tasks.

In recent years, vision-based deep learning has shown strong potential in automated plant phenotype analysis and quality evaluation. Deep neural networks have been successfully applied to tasks such as safflower germplasm classification, demonstrating high accuracy under field conditions (Van et al., 2025). For fine-grained tasks like filament-level analysis, CNN models designed for unstructured environments have also shown promising results (Chen et al., 2024a). However, the visual complexity of safflower filaments—including subtle textures, shape variability, and frequent overlaps—poses challenges for real-time and precise classification. These factors often lead to a trade-off between accuracy and inference speed, limiting the practical deployment of current models in large-scale agricultural systems. To improve feature extraction and reduce model complexity, several studies have proposed lightweight modifications to core network modules. For example, Wang and Liu (2024) introduced a GhostConv-based variant of the C2f module, which significantly reduced parameter counts while maintaining detection accuracy. In another study, Wang et al. (2025) proposed a pyramid-style C2f structure to enhance multi-scale feature learning and improve computational efficiency. Meanwhile, efforts have also focused on integrating attention mechanisms into feature fusion designs. Miao et al. (2025) developed a dynamic convolution and spatial attention architecture to better capture both local and global semantics. In the field of crop quality grading, Zhao et al. (2024) presented an attention-enhanced classification framework that substantially improved prediction accuracy in complex agricultural scenarios. In addition, lightweight detection heads have been proposed to reduce computation costs during inference, particularly for use in embedded or resource-constrained agricultural environments (Qing et al., 2024; Wang et al., 2025). Despite these advancements, existing approaches remain focused on general object detection and often fail to address the fine-grained structural characteristics of safflower filaments. Attention modules tend to emphasize global context while overlooking local morphological cues such as edge continuity and curvature. Furthermore, decoupled classification heads may introduce information loss, reducing model reliability in detail-sensitive tasks. These limitations underscore the need for a model that is both lightweight and capable of fine-structure modeling, specifically tailored for real-time filament-level classification in agricultural applications.

Beyond CNN- and attention-based lightweight designs, recent alternative paradigms have emerged. For hyperspectral scenarios, SpectralMamba (Yao et al., 2024) adopts a state-space-model backbone that combines gated spatial–spectral interaction with efficient sequential modeling to balance accuracy and efficiency. Complementarily, SPECIAL (Pang et al., 2025) presents a CLIP-based zero-shot pipeline that interpolates HSI into RGB bands to obtain pseudo-labels and then refines them via noisy-label learning. While our study targets RGB-based safflower filament grading under a supervised setting, these principles—efficient state–space feature interaction and label-efficient supervision—outline promising directions for future lightweight classification systems in spectral or multi-modal agricultural applications.

To address these challenges, this study proposes CNATNet, a lightweight convolution-attention hybrid model designed for efficient and accurate safflower filament classification. CNATNet integrates multi-branch feature extraction, attention-enhanced feature fusion, and lightweight prediction modules to ensure high classification accuracy while meeting practical requirements for low latency and limited computational resources. Specifically, CNATNet is tailored to the morphological characteristics of safflower filaments, adopting a coarse-to-fine recognition strategy that progressively refines feature representations from cluster-level patterns to filament-level details. The model architecture builds upon established lightweight designs, including MobileNetV2 (Sandler et al., 2018), MobileNetV3 (Howard et al., 2019), and GhostViT (Cao et al., 2024), achieving an effective balance between recognition performance and computational efficiency. On this basis, CNATNet introduces the following key contributions:

1. C2S2 convolution module: To enhance feature extraction efficiency, a novel C2S2 module is designed by partitioning feature channels into multiple lightweight parallel branches, enabling efficient convolution operations. The cascaded connection structure further strengthens the module’s capacity to capture filament directionality, preserve curvature continuity, and maintain edge integrity. This lightweight design allows the network to effectively represent fine-grained morphological features critical for accurate filament classification under complex visual conditions.
2. AnC2f attention module: To better cope with the fine-grained texture and overlapping structures of safflower, an attention mechanism is incorporated. AnC2f enhances the model’s sensitivity to key spatial regions and multi-scale cues by gradually stacking lightweight attention modules in the residual fusion path. This design improves the network’s ability to capture subtle structural changes, enabling more accurate distinction between high-quality and ordinary-grade filaments.
3. DWClassify lightweight classification head: By decoupling spatial and channel features through depthwise separable convolution, DWClassify substantially reduces computational complexity without sacrificing prediction accuracy. This ensures that the model can achieve real-time inference not only on high-performance GPUs but also on resource-constrained embedded platforms, reinforcing its lightweight nature.
4. Deployment on Jetson Orin Nano: The optimized CNATNet model was deployed on the Jetson Orin Nano, a high-performance yet power-efficient embedded platform. Real-time inference was achieved for safflower filament and cluster classification at 63 FPS under 15 W, maintaining high accuracy under constrained computational budgets. These results confirm that CNATNet is not only accurate but also lightweight and deployment-ready, providing a practical and scalable solution for intelligent plant quality assessment in modern agriculture.

## 2 Materials and methods

### 2.1 Data collection

Xinjiang is the largest safflower-producing region in China, with its favorable climate and soil conditions contributing to filaments that are superior in color, texture, and bioactive compound content compared to those from other regions. The experimental sites for this study were located in the main safflower production areas of Changji Hui Autonomous Prefecture, including Changji City, Manas County, and Hutubi County. The collected samples from these areas were characterized by vivid coloration, fine and intact filament structure, and high medicinal and economic value.

**Figure 1:**
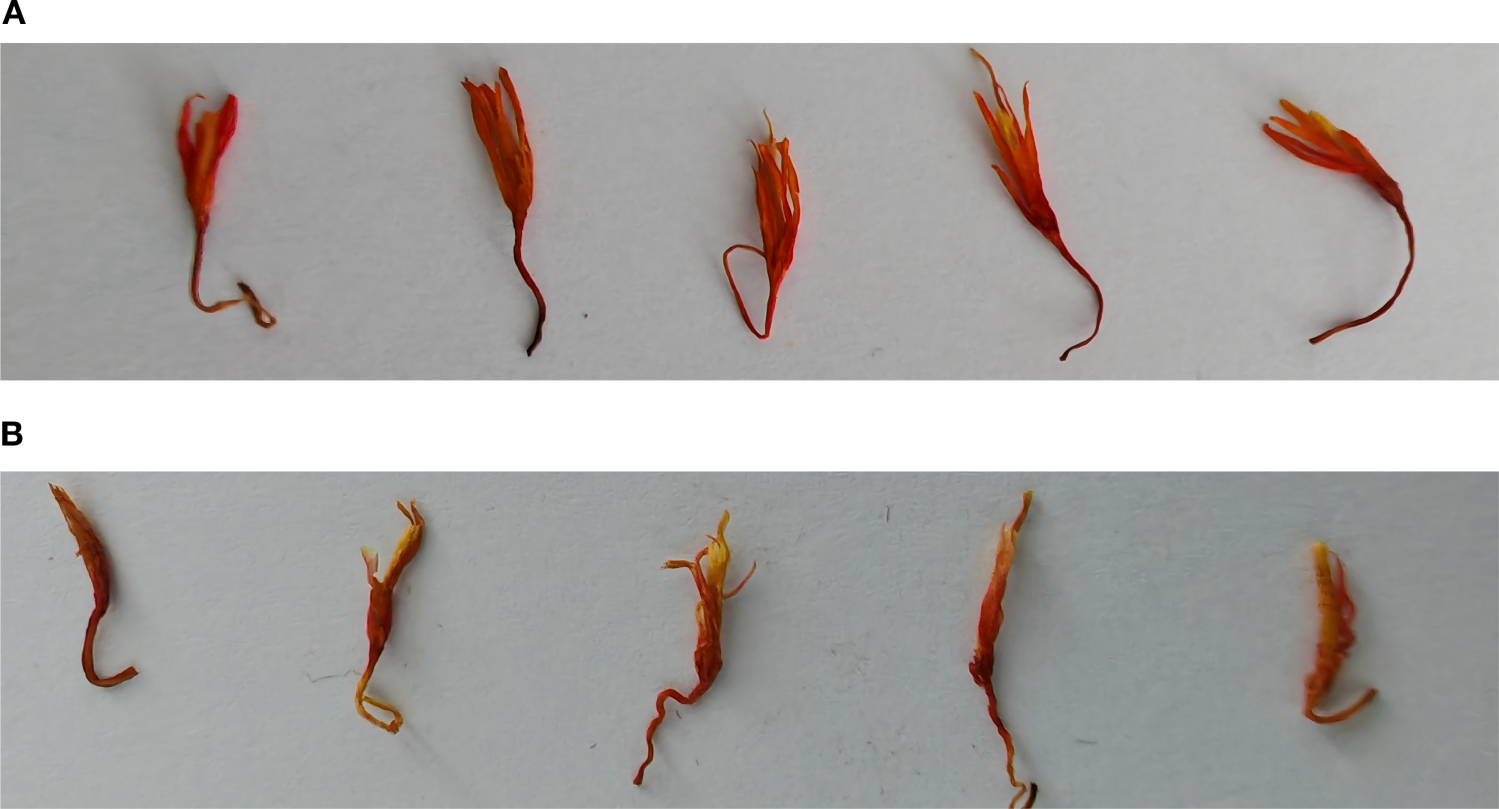
Visual comparison of safflower filaments across different quality grades. **(A)** High-quality *Carthamus tinctorius* (L.) filament. **(B)** Common-quality *Carthamus tinctorius* (L.) filament.

Traditionally, safflower classification relies on quantifying active constituents such as safflower yellow pigment. However, these methods are time-consuming and involve complex chemical operations, making them unsuitable for real-time classification or automated sorting systems. To meet the demands of automation, this study adopts a visual classification standard based on the appearance of safflower filaments. By combining field observations and expert feedback from local growers, the filaments were categorized into two major quality classes: premium-grade filaments suitable for medicinal applications, and regular-grade filaments intended for auxiliary uses. Premium-grade filaments are defined by their bright color, compact structure, and high fiber integrity, making them ideal for clinical or health-related purposes. In contrast, regular-grade filaments exhibit dimmer coloration, inconsistent thickness, and lower fiber quality, and are better suited for daily health care, soaking, or pigment extraction. This classification standard is practical for both manual annotation and automated model training and effectively captures intrinsic quality differences. **Figure 1** presents representative examples of premium-grade and regular-grade single safflower filaments, illustrating their visual distinctions in color, texture, and structural integrity.

From June to August 2024, safflower filament images were acquired across multiple plantation sites in Changji Prefecture using a mobile imaging setup equipped with an iPhone 14 Pro Max. All samples were selected from post-harvest and naturally air-dried filaments to ensure consistency with real-world processing conditions. To enhance dataset diversity and improve the robustness of model generalization, images were acquired under diverse environmental conditions. Specifically, the dataset was constructed by capturing images across various camera angles, illumination levels, and background complexities. The acquisition scenarios included:

(i) outdoor environments under natural daylight; (ii) indoor settings with diffuse artificial lighting; (iii) backgrounds exhibiting different levels of clutter and occlusion.

A total of 5,800 images were acquired at a resolution of 3840×2160 pixels and saved in PNG format to ensure lossless preservation. The dataset comprises both scattered single filaments and densely stacked floral clusters. Image acquisition was performed at various distances, ranging from close-up views (*<*0.15 m) to medium-to-long ranges (*>*0.15 m), thereby enhancing the robustness and generalization capability of the proposed classification model.

### 2.2 Dataset creation and annotation

**Figure 2:**
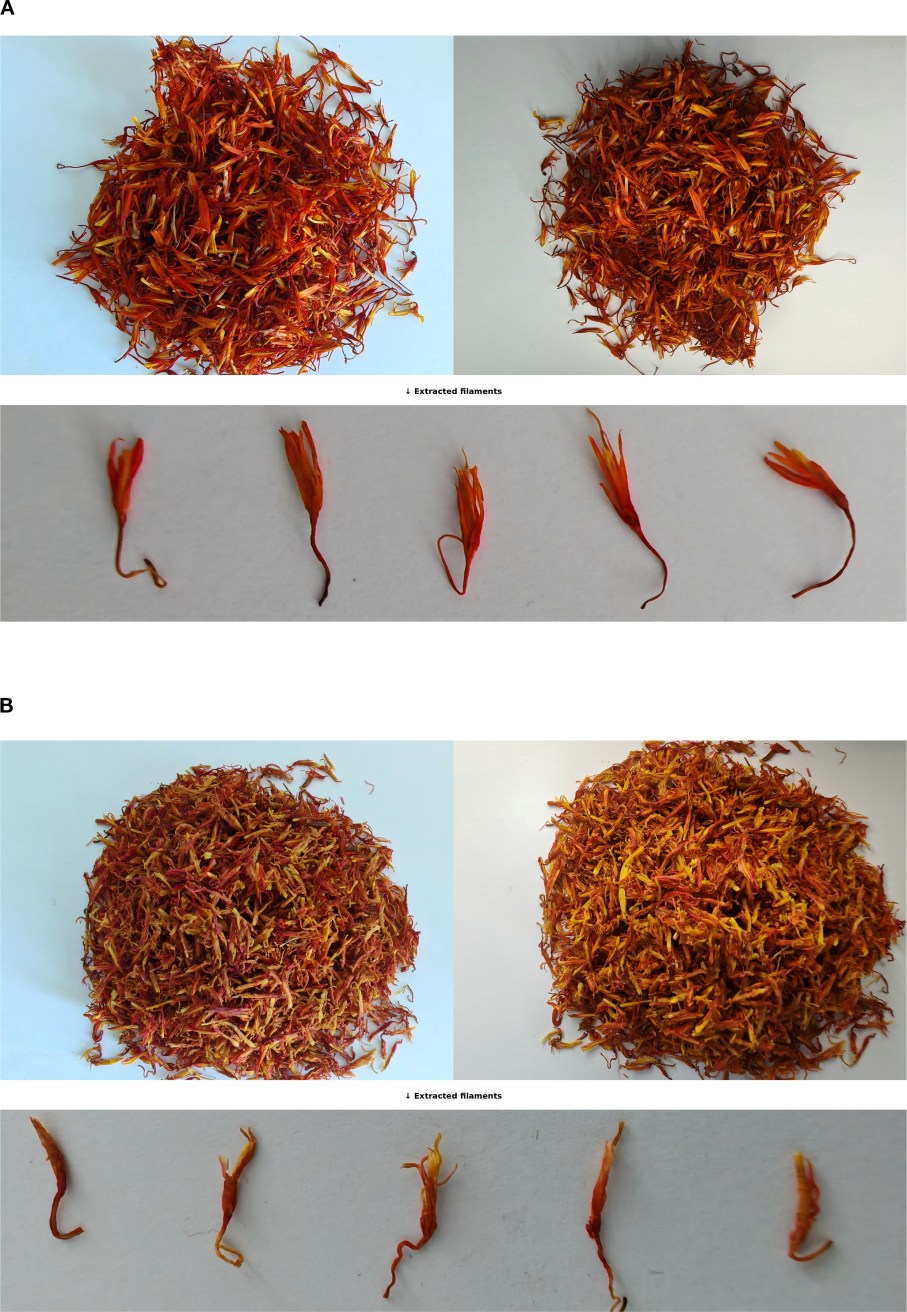
Visual comparison of safflower filaments under different quality grades and lighting conditions. **(A)** High-quality sample. **(B)** Common-quality sample.

To ensure annotation accuracy prior to model training, all original safflower filament images were labeled using the Roboflow platform. Each image was annotated with a single instance representing either a premium-grade or regular-grade filament. The two categories were respectively labeled as *“CT Premium”* and *“CT Normal”*. Each image contains only one target object, either an isolated filament or a dense filament cluster, assigned with the appropriate class label. Considering the influence of environmental variation—such as fluctuating lighting and complex backgrounds—this study adopts a binary classification strategy based on single-instance images. The model directly learns quality classification from global visual features of individual filaments. This strategy enables the model to focus on overall filament appearance, thereby improving classification accuracy and robustness under challenging real-world conditions. To visually illustrate the environmental variations considered in the proposed classification strategy, representative filament samples captured under different lighting conditions are shown in **Figure 2**. For each quality grade, the left panel presents filaments photographed under natural daylight, while the right panel corresponds to images captured with diffuse artificial lighting. Additionally, the lower row displays individual filaments extracted from the bulk samples, serving as single-instance inputs for classification.

The safflower filament dataset used in this study was constructed through systematic image acquisition under diverse environmental conditions. To ensure a comprehensive representation of real-world scenarios, images were captured across multiple variables, including filament quantity (single or multiple filaments), shooting distance (close-up or distant view), illumination condition (natural or supplementary lighting), camera angle, and background complexity. Owing to natural variations in field cultivation and post-harvest processing, the collected dataset exhibits differences in filament quality, structure, and visual appearance. A total of 5,800 high-resolution images (3840×2160 pixels) were acquired, covering both premium-grade and common-grade safflower filaments. The detailed class distribution is summarized in **Table 1**.

**Table 1:** Class distribution of the safflower dataset under various acquisition conditions.

Scenario	Category	Premium	Normal	Total
Filament quantity	Single filaments	1650	1350	3000
	Multiple filaments	1450	1350	2800
Shooting distance	Close-up view	1750	1550	3300
	Distant view	1350	1150	2500
Lighting condition	Natural lighting	1900	1600	3500
	Supplementary lighting	1200	1100	2300
Camera angle	Frontal view	1600	1400	3000
	Multi-angle view	1500	1300	2800
Background complexity	Clean background	1800	1500	3300
	Cluttered background	900	800	1700
	Occluded background	800	700	1500

**Figure 3:**
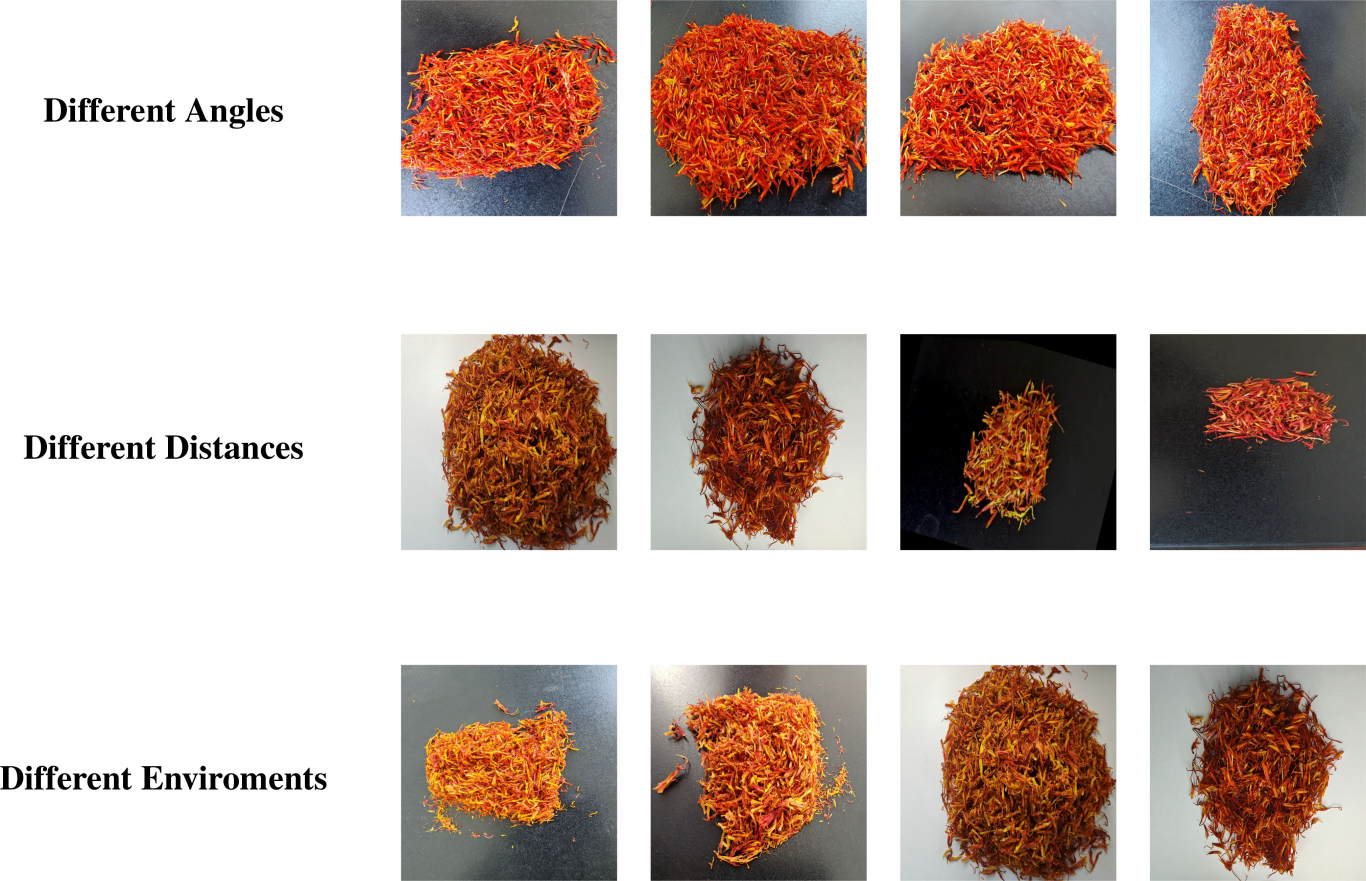
Visual examples of safflower captured under different acquisition conditions.

**Figure 4:**
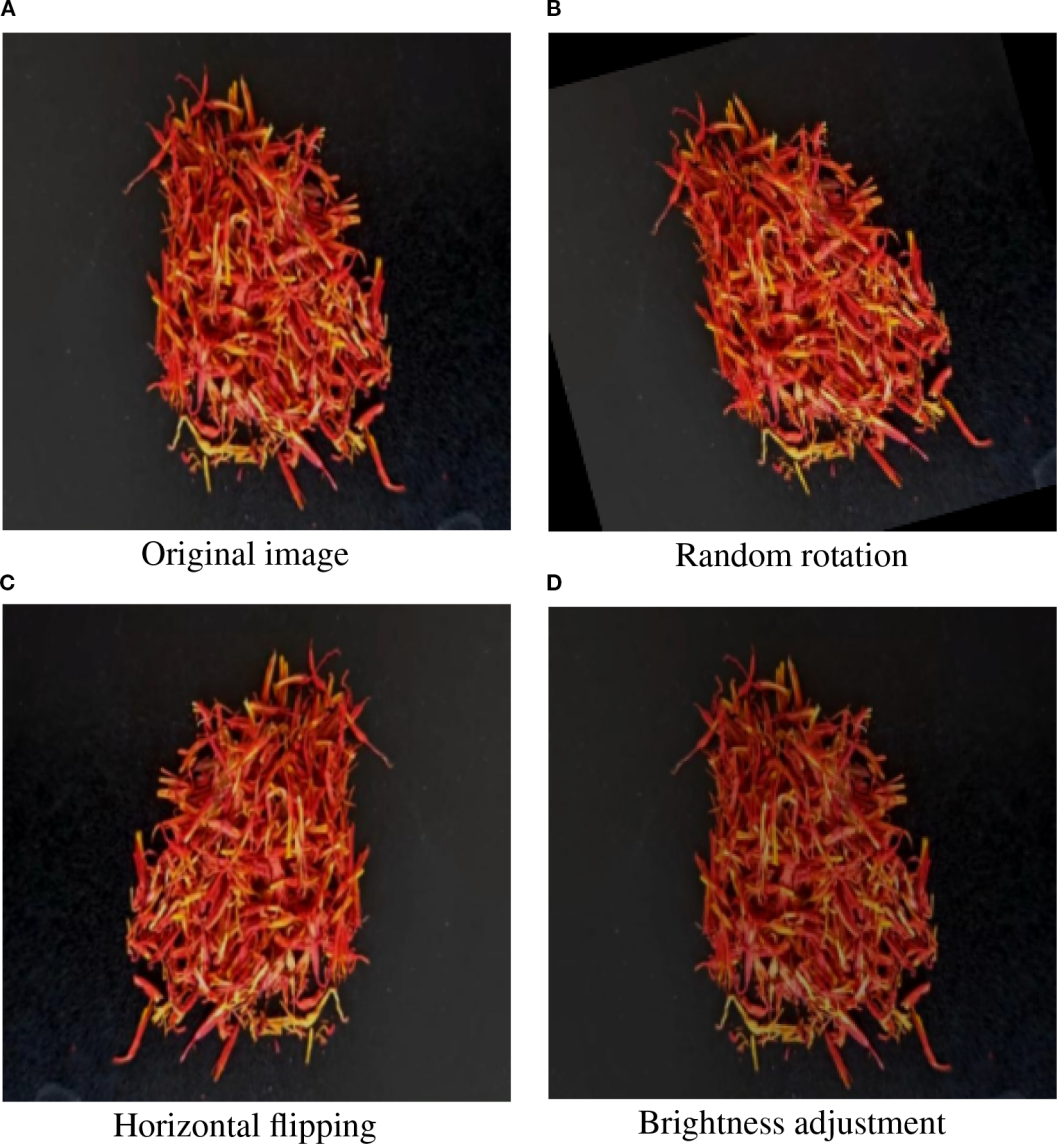
Representative examples of augmented safflower filament images. **(A)** Original image, **(B)** random rotation, **(C)** Horizontal flipping, **(D)** Brightness adjustment.

To support downstream model training and evaluation, the full dataset was randomly partitioned into training, validation, and testing subsets at a ratio of 7:2:1. The class distribution was maintained consistently across all subsets to ensure balanced data quality and enable fair performance comparisons. The collected images cover diverse acquisition conditions, including variations in shooting angles, shot distances, and environmental complexities, to enhance model robustness and generalization, as illustrated in **Figure 3**. To mitigate overfitting and reduce sensitivity to class distribution bias, multiple data augmentation techniques were applied during training. Specifically, random rotation, horizontal flipping, brightness adjustment, and affine transformations were employed to simulate variations in angle, lighting, and background complexity. These augmentations not only enrich the diversity of training samples but also enhance the model’s generalization capability under challenging conditions. Representative examples of these augmentation strategies are illustrated in **Figure 4**.

### 2.3 Model architecture

#### 2.3.1 CNATNet framework overview

**Figure 5:**
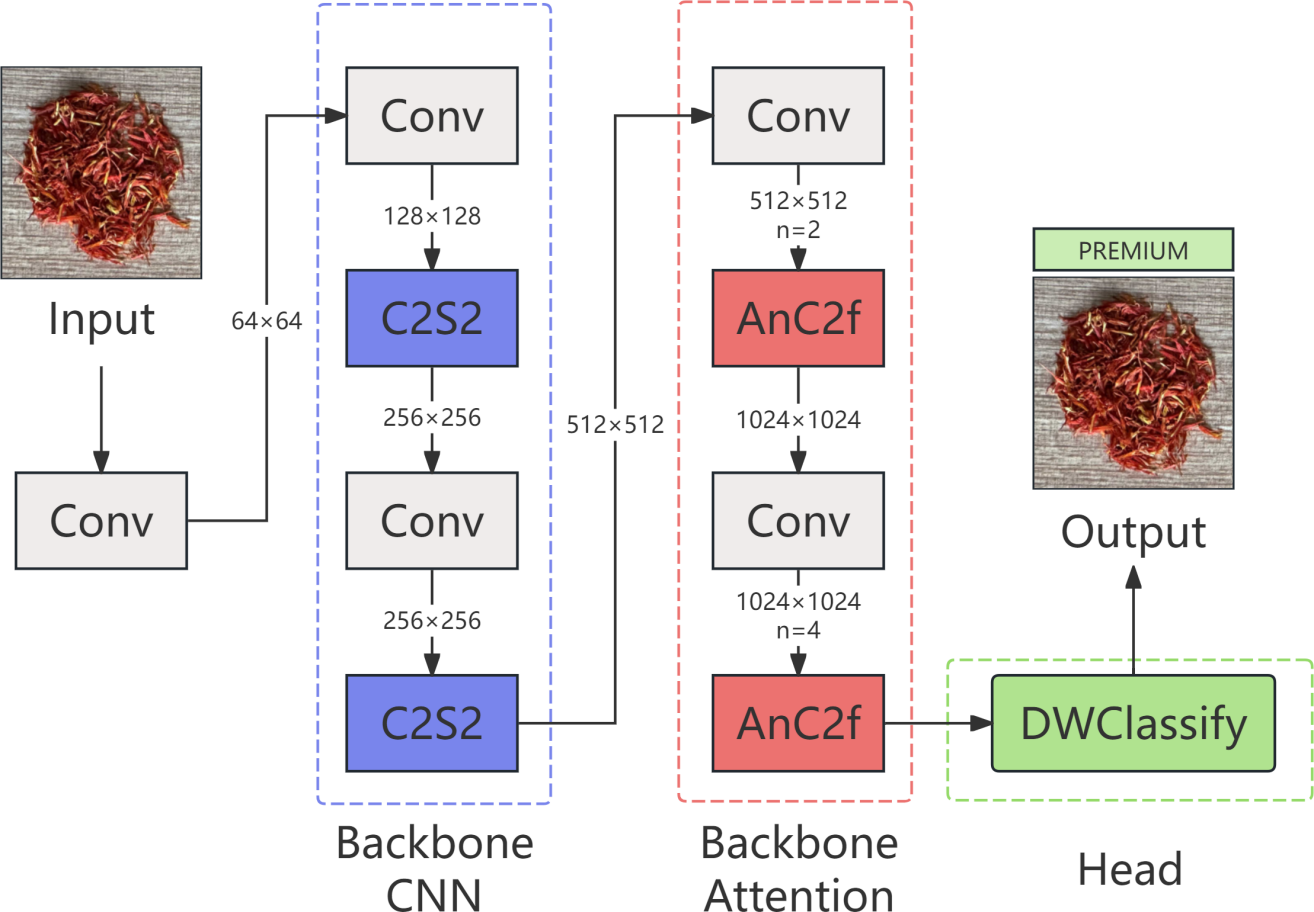
CNATNet structure diagram.

CNATNet is a hybrid network that integrates convolutional neural networks with attention mechanisms. It consists of two main components: a backbone for multi-scale feature extraction and fusion, and a classification head for safflower filament quality prediction. As illustrated in **Figure 5**, the architecture integrates both convolutional and attention-based modules across feature extraction and classification stages. Specifically, the backbone incorporates convolution-centric C2S2 modules and attention-enhanced AnC2f modules, forming a synergistic structure that leverages the strengths of both paradigms. The C2S2 module focuses on efficient representation learning with reduced computational cost (Chen et al., 2024b; Liu et al., 2024), while the AnC2f module enhances spatial attention and multi-scale perception through stacked attention blocks (Li et al., 2021; Lv et al., 2022). For final prediction, CNATNet employs a lightweight DWClassify head based on depthwise separable convolutions to minimize parameter count and optimize inference speed (Dwika Hefni Al-Fahsi et al., 2024; Gao et al., 2021).

#### 2.3.2 C2S2: cascaded split-and-concatenate structure

**Figure 6:**
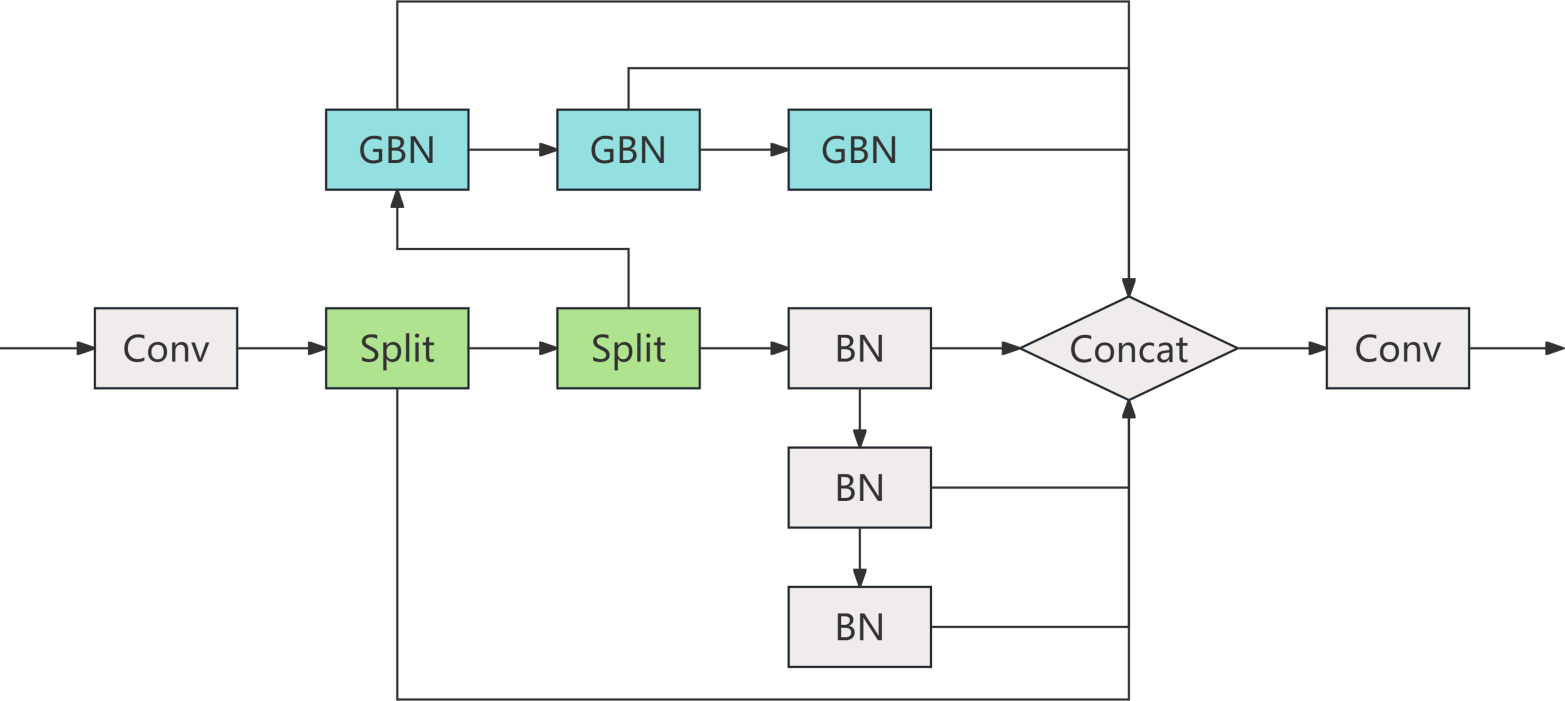
Internal architecture of the C2S2 module.

C2S2 is a lightweight convolutional feature extractor designed for early-stage processing within CNATNet. It starts with a standard convolution to extract base-level features, followed by a channel-wise split into two parallel branches. Each branch processes features through stacked GhostBottleneck or Bottleneck layers to enhance representational capacity while reducing parameter overhead (Huang and Wang, 2025; Yu and Zhou, 2023). The internal architecture of C2S2 is illustrated in **Figure 6**, which details the feature flow, dual-branch operations, and lightweight convolutional structure.

After local refinement in each branch, a bottleneck layer compresses channel dimensions to extract key features, which are then concatenated across branches to form the unified output. Mathematically, the feature fusion process of C2S2 can be expressed as (Equation 1):


(1) $$F_{C2S2}=\text{Concat}\left(f_{1}\left(X_{1}\right),f_{2}\left(X_{2}\right)\right)$$

where *X*_1_ and *X*_2_ represent the channel-wise partitions of the input feature map *X*, which are independently processed by the branch-specific transformation functions *f*_1_(·) and *f*_2_(·), respectively. The outputs of these branches are concatenated along the channel dimension by the Concat(·) operation, resulting in the unified feature representation *F_C_*_2_ *_S_*_2_.

The parameter complexity of C2S2 is quantified in Equation 2:


(2) $$P_{C2S2}=\sum_{i=1}^{2}\left(C_{split}\times K\times K\times C_{split}\right)+C_{merge}\times C_{out}$$

where *C_split_* denotes the number of channels assigned to each branch after splitting, *K* represents the convolution kernel size, *C_merge_* refers to the number of channels in the fusion (bottleneck) layer, and *C_out_* is the output channel number of the C2S2 module. The first summation term calculates the cumulative parameter count of the dual-branch convolutions, each operating within its respective channel partition, while the final term accounts for the parameters introduced by the merging operation that recombines the branch-specific features.

By leveraging its parallel structure, C2S2 achieves a balanced trade-off among computational efficiency, feature diversity, and structural consistency, making it particularly well-suited for processing safflower filament images with directional textures and densely packed patterns (Lau et al., 2024; Zhu et al., 2024). This design ensures robust feature extraction while maintaining low computational overhead, which is critical for practical deployment in resource-constrained environments.

#### 2.3.3 AnC2f: attention-enhanced cross-stage fusion

**Figure 7:**
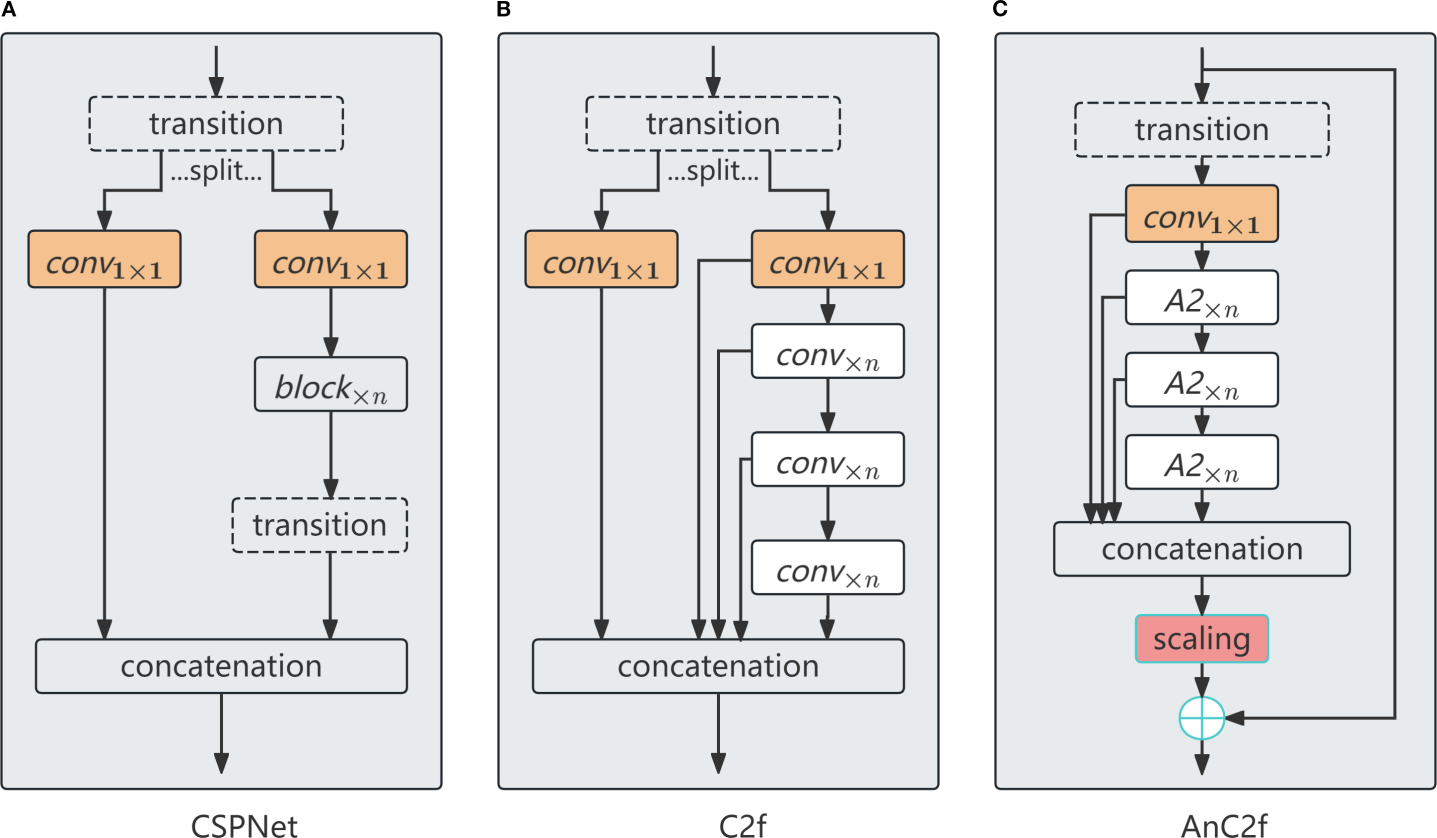
Structural comparison among CSPNet **(A)**, C2f **(B)**, and the proposed AnC2f **(C)**.

AnC2f is an attention-driven fusion module inspired by the C2f architecture from YOLOv8 and the original CSPNet structure (Varghese and M, 2024; Wang et al., 2019). It enhances cross-stage learning by injecting multiple stacked Attention Blocks (ABlocks) into the fusion process. The structural details of the AnC2f module are depicted in **Figure 7**. As shown, AnC2f retains the dual-path feature flow of C2f, where the input features are split into two branches: a shortcut branch for direct feature propagation, and a main branch for progressive feature refinement.

In the main processing branch, stacked Attention Blocks (ABlocks) are applied to modulate the input features through spatially adaptive weighting. This modulation process is formulated as (Equation 3):


(3) $$F_{AnC2f}=\sigma \left(\text{Con}v\left(F_{in}\right)\right)⊙F_{in}$$

where *F_in_* denotes the input feature map, Conv(·) represents a 1 × 1 convolutional layer that generates attention weights, *σ*(·) is the Sigmoid activation function ensuring the attention weights are bounded between 0 and 1, and ⊙ denotes element-wise multiplication, enabling spatial modulation of *F_in_*.

To preserve the original information flow, AnC2f incorporates a shortcut branch that directly propagates the input features without attention modulation. The outputs of the main and shortcut branches are then fused through element-wise addition, as defined in (Equation 4):


(4) $$F_{AnC2fOut}=F_{shortcut}+F_{AnC2f}$$

where *F_shortcut_* refers to the identity feature path, facilitating information retention and gradient propagation, while *F_AnC_*_2_ *_f_* represents the attention-refined features from the main branch. This residual fusion mechanism ensures that the enhanced attention features are complemented by the original unaltered information, leading to richer and more robust representations.

By seamlessly integrating attention-driven enhancement with lightweight computational design, AnC2f effectively improves multiscale feature learning, contributing to the overall performance of CNATNet while maintaining high efficiency.

AnC2f divides input features into parallel paths, applying convolutional and attention operations independently before recombining them via residual connections. Attention blocks are stacked in parallel to progressively improve the model’s sensitivity to important spatial regions and morphological patterns (Ding et al., 2021; Yin et al., 2024). This enables the network to more effectively capture fine-grained structural details characteristic of safflower filaments.

#### 2.3.4 DWClassify: lightweight classification head

DWClassify functions as the final classification head in CNATNet, aiming to deliver accurate predictions with minimal computational overhead. To this end, DWClassify adopts a depthwise separable convolutional structure, which effectively decouples spatial and channel-wise feature extraction. This design choice significantly reduces both the parameter count and computational complexity compared to conventional convolutional layers (Dwika Hefni Al-Fahsi et al., 2024; Wang et al., 2023a).

The parameter complexity of DWClassify is quantified as (Equation 5):


(5) $$P_{DW}=C_{in}\times K\times K+C_{in}\times C_{out}$$

where *C_in_* and *C_out_* denote the input and output channel dimensions, respectively, and *K* represents the kernel size of the depthwise convolution. Specifically, the first term *C_in_* × *K* × *K* corresponds to the parameters of the depthwise convolution, which performs spatial filtering independently on each input channel. The second term *C_in_* × *C_out_* accounts for the pointwise convolution parameters, responsible for inter-channel feature aggregation via 1 × 1 convolutions.

In addition to parameter reduction, DWClassify exhibits high computational efficiency. The floating-point operations (FLOPs) required for inference are estimated by (Equation 6):


(6) $$FLOPs_{DW}=H\times W\times \left(C_{in}\times K\times K+C_{in}\times C_{out}\right)$$

where *H* and *W* denote the spatial dimensions of the input feature map. The multiplication with *H* × *W* accounts for the per-pixel computation cost across the entire feature map. Similar to the parameter calculation, the first term reflects the spatial filtering cost of the depthwise convolution, while the second term represents the channel-wise aggregation cost incurred by the pointwise convolution.

**Figure 8:**
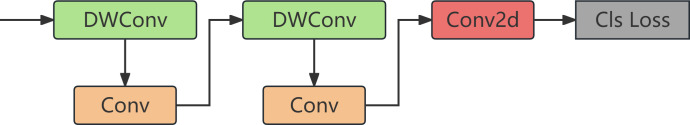
Architecture of the DWClassify module.

The architectural details of DWClassify are illustrated in **Figure 8**. By decoupling spatial and channel-wise operations, DWClassify achieves an optimal balance between model compactness and predictive accuracy. This lightweight design not only enhances inference speed but also ensures seamless deployment in resource-constrained environments such as embedded devices and mobile platforms.

## 3 Results and analysis

### 3.1 Experimental setup and evaluation metrics

#### 3.1.1 Experimental environment and parameters

All experiments were conducted on a workstation equipped with an NVIDIA RTX 3070 Ti GPU. The model was implemented using the PyTorch 1.12 framework under a Windows environment. For optimization, the Adam optimizer was employed with a learning rate of 0.001. The training process utilized a batch size of 32 and was conducted for a total of 300 epochs. The specific configuration details are summarized in **Table 2**.

**Table 2:** Experimental environment configuration.

Component	Configuration
GPU	NVIDIA RTX 3070 Ti
Framework	PyTorch 1.12
Optimizer	Adam
Learning Rate	0.001
Batch Size	32
Epochs	300

#### 3.1.2 Evaluation metrics

To comprehensively evaluate the performance of the proposed CNATNet model, this study adopts four core metrics: floating point operations (FLOPs), number of parameters (Params), accuracy (ACC), and latency. These metrics jointly assess the computational efficiency, model complexity, prediction precision, and real-time inference capability of the network.

FLOPs measure the computational complexity required for a single forward pass, indicating the model’s resource consumption. The total FLOPs are computed based on the number of operations performed per spatial location and aggregated over all feature maps, as formulated in Equation 7:


(7) $$\text{FLOPs}=\sum_{l=1}^{L}H_{l}\times W_{l}\times C_{in}^{\left(l\right)}\times K\times K\times C_{out}^{\left(l\right)}$$

where *L* denotes the total number of convolutional layers, *H_l_* and *W_l_* represent the spatial dimensions of the *l*-th layer’s feature map, $C_{in}^{\left(l\right)}$ and $C_{out}^{\left(l\right)}$ are the input and output channel numbers, and *K* is the kernel size.

Params refer to the total count of learnable parameters within the model, directly reflecting its memory footprint. The calculation is expressed in Equation 8:


(8) $$\text{Params}=\sum_{l=1}^{L}C_{in}^{\left(l\right)}\times K\times K\times C_{out}^{\left(l\right)}+C_{out}^{\left(l\right)}$$

where the first term accounts for convolutional weights and the second term represents biases.

Accuracy (ACC) measures the ratio of correctly classified samples to the total number of samples in the test set, providing an intuitive evaluation of the model’s classification capability. The formula is shown in Equation 9:


(9) $$\text{ACC}=\frac{TP+TN}{TP+TN+FP+FN}$$

where *TP*, *TN*, *FP*, and *FN* denote true positives, true negatives, false positives, and false negatives, respectively.

Latency quantifies the average time taken to process a single input image during inference. This metric reflects the practical deployment capability of the model, particularly in real-time scenarios. The latency is defined in Equation 10:


(10) $$\text{Latency}=\frac{T_{total}}{N_{samples}}$$

where *T_total_* represents the total inference time across all test samples, and *N_samples_* is the number of test samples.

### 3.2 Experimental results and analysis

To comprehensively evaluate the proposed model’s effectiveness in real-world safflower sorting scenarios, we divided the classification task into two distinct levels: cluster classification and monomer classification. The cluster-level task simulates coarsegrained recognition of densely packed safflower clusters, where the model must make a global judgment based on group-level visual cues. This setup reflects typical conditions in automated harvesting or packaging lines, where safflower bundles are processed in bulk. In contrast, the monomer-level task focuses on fine-grained classification of individual filaments, emphasizing subtle morphological differences such as color, curvature, and integrity. This setting aligns with higher-precision quality control scenarios, such as pharmaceutical sorting or premium product filtering. By evaluating both levels independently, we aim to demonstrate the robustness and generalizability of CNATNet across varied granularities of visual complexity.

#### 3.2.1 Cluster classification results

**Figure 9:**
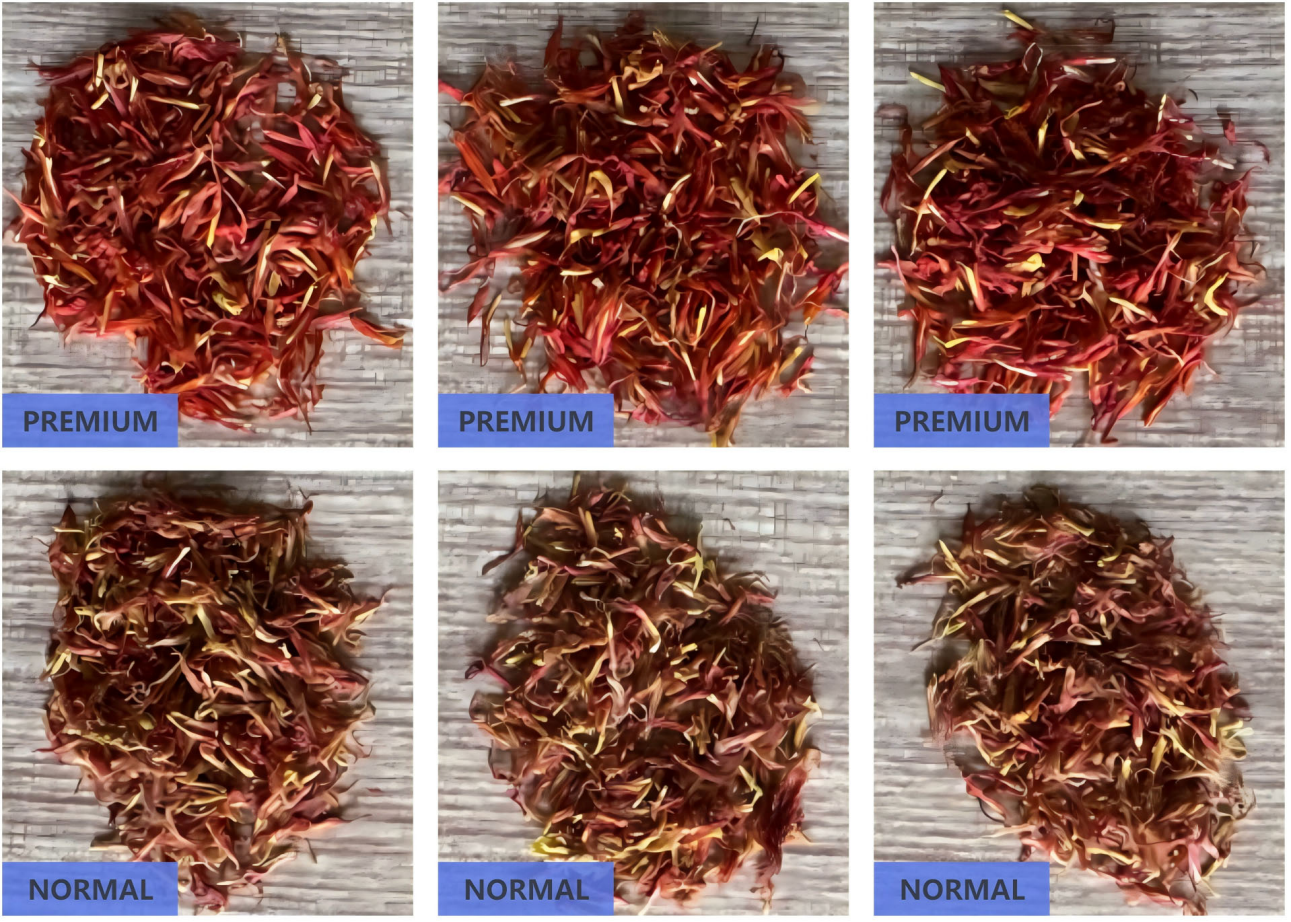
Cluster-level classification visualization with CNATNet.

To evaluate the effectiveness of different models in identifying the overall quality of densely packed safflower clusters, we formulated a cluster-level classification task. In this setting, the input images consist of multiple overlapping filaments, simulating the typical appearance of harvested safflower in agricultural processing lines. A comprehensive comparison was conducted across a wide set of models, including CNATNet, YOLOv5s, YOLOX (Ge et al., 2021), DAMO-YOLO (Xu et al., 2022b), PP-YOLOE (Xu et al., 2022a), YOLOv6 (Li et al., 2022), YOLOv7 (Wang et al., 2023b), YOLOv8n/s/m, YOLOv10n/s/m (Wang et al., 2024), YOLOv11n/s/m, RT-DETRv2s (Lv et al., 2024), and RF-DETR-B. The complete quantitative results are summarized in **Table 3**. The visual detection results of CNATNet for cluster classification are shown in **Figure 9**.

**Table 3:** Quantitative results of the cluster classification experiment.

Model	FLOPs (B)	Params (M)	Accuracy (%)	Latency (ms)
CNATNet	4.6	9.8	98.6	1.9
YOLOv5s	1.7	2.4	85.2	1.5
YOLOXs	–	–	91.4	1.5
DAMO-YOLO-1	–	–	84.4	1.3
PP-YOLOE+s	–	–	85.6	1.5
YOLOv6-3.0n	–	–	85.4	1.2
YOLOv7l	–	–	93.5	4.2
YOLOv8n	0.5	2.7	85.0	1.1
YOLOv8s	1.7	6.4	88.0	1.5
YOLOv8m	5.3	17.0	91.3	3.3
YOLOv10n	0.5	1.6	89.4	1.2
YOLOv10s	1.7	5.8	91.6	1.6
YOLOv10m	5.1	11.7	93.5	2.9
YOLO11n	0.5	1.6	89.4	1.1
YOLOv11s	1.6	5.5	92.2	1.4
YOLO11m	5.1	10.4	93.9	2.1
RT-DETRv2s	–	20.0	86.4	2.3
RF-DETR-B	–	19.7	95.8	2.2

As shown in the table, CNATNet achieved the highest classification accuracy (98.6%) while maintaining low latency (1.9 ms) and moderate model size (9.8 M parameters, 4.6 B FLOPs), highlighting its superior balance between performance and computational efficiency. In contrast, lightweight variants such as YOLOv8n and YOLOv10n offered faster inference but at the expense of accuracy, while larger-scale models such as YOLOv7l and YOLOv11m provided competitive accuracy but with considerably higher latency. Transformer-based approaches (RT-DETRv2s and RF-DETR-B) demonstrated stronger representational capacity, with RF-DETR-B achieving the second-highest accuracy (95.8%) but requiring higher model complexity.

It should be noted that for several comparative models, including YOLOX, DAMO-YOLO, PP-YOLOE, YOLOv6, YOLOv7, RT-DETRv2, and RF-DETR, FLOPs and parameter counts were not reported in their original publications or repositories, and are thus marked as “–” in **Table 3**. Since accuracy and latency are available for all methods, the comparative evaluation remains fair and informative.

#### 3.2.2 Monomer classification results

**Figure 10:**
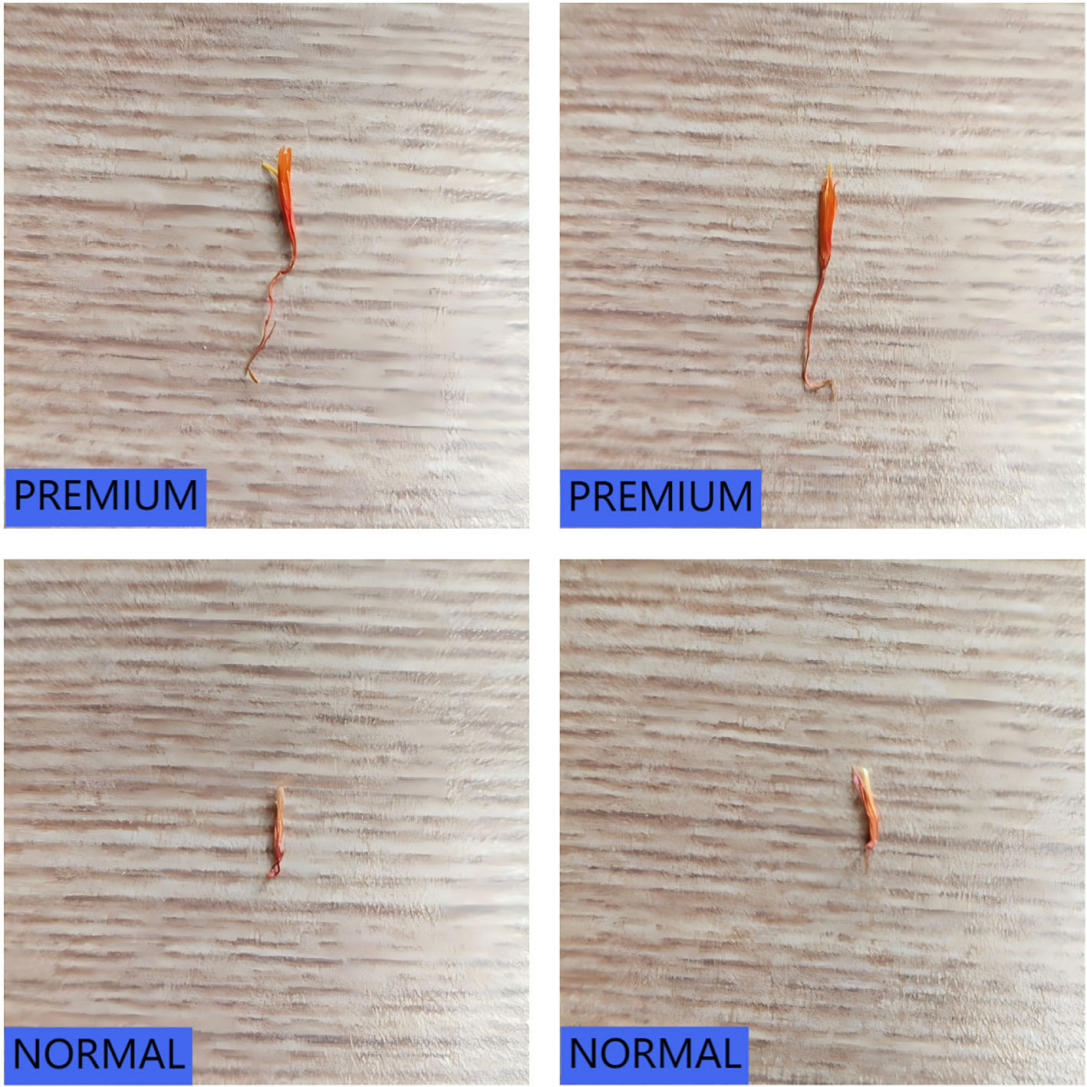
Filament-level classification visualization with CNATNet.

To further evaluate the models on fine-grained morphological recognition, a filament-level classification task was formulated. In this setting, each input image contained a single isolated safflower filament, requiring the models to identify subtle visual cues such as color, curvature, and integrity. A comprehensive comparison was performed across CNATNet, YOLOv5s, YOLOX (Ge et al., 2021), DAMO-YOLO (Xu et al., 2022b), PP-YOLOE (Xu et al., 2022a), YOLOv6 (Li et al., 2022), YOLOv7 (Wang et al., 2023b), YOLOv8n/s/m, YOLOv10n/s/m (Wang et al., 2024), YOLOv11n/s/m, RT-DETRv2s (Lv et al., 2024), and RF-DETR-B, all trained and tested under identical conditions. The complete quantitative results are reported in **Table 4**. The visual detection results of CNATNet for monomer classification are shown in **Figure 10**.

**Table 4:** Quantitative results of the filament classification experiment.

Model	FLOPs (B)	Params (M)	Accuracy (%)	Latency (ms)
CNATNet	4.6	9.8	95.6	1.9
YOLOv5s	1.7	2.4	82.6	1.5
YOLOXs	–	–	88.7	1.5
DAMO-YOLO-1	–	–	81.5	1.3
PP-YOLOE+s	–	–	82.9	1.5
YOLOv6-3.0n	–	–	83.0	1.2
YOLOv7l	–	–	91.0	4.2
YOLOv8n	0.5	2.7	82.3	1.1
YOLOv8s	1.7	6.4	85.4	1.5
YOLOv8m	5.3	17.0	88.6	3.3
YOLOv10n	0.5	1.6	86.5	1.2
YOLOv10s	1.7	5.8	89.2	1.6
YOLOv10m	5.1	11.7	91.0	2.9
YOLO11n	0.5	1.6	85.8	1.1
YOLOv11s	1.6	5.5	88.3	1.4
YOLO11m	5.1	10.4	86.4	2.1
RT-DETRv2s	–	20.0	83.8	2.3
RF-DETR-B	–	19.7	92.1	2.2

As shown in the table, CNATNet achieved the highest accuracy (95.6%) with a low inference latency (1.9 ms) and moderate model complexity (9.8 M parameters, 4.6 B FLOPs), confirming its ability to balance efficiency and precision in fine-grained filament recognition. In contrast, lightweight models such as YOLOv8n and YOLOv10n delivered faster inference but suffered noticeable accuracy drops, whereas larger models like YOLOv7l and YOLOv10m achieved higher accuracy at the cost of increased latency. Transformer-based architectures exhibited strong representational power, with RF-DETR-B reaching the second-highest accuracy (92.1%) but with substantially larger model size and computational requirements.

It should be emphasized that for several comparative models, including YOLOX, DAMO-YOLO, PP-YOLOE, YOLOv6, YOLOv7, RTDETRv2, and RF-DETR, FLOPs and parameter counts were not reported in their original papers or official repositories. These values are therefore omitted (“–”) in **Table 4**. Nevertheless, since both accuracy and latency are consistently available, the comparative evaluation remains comprehensive and fair.

### 3.3 Ablation study on structural components

An ablation study was conducted to investigate the individual and joint contributions of the C2S2, AnC2f, and DWClassify modules to the overall performance of CNATNet, the results are shown in **Table 5**. The baseline model (M1) was constructed by removing all three proposed modules, employing a conventional convolutional residual block as the backbone, a simplified C2f neck without attention mechanisms, and a dense convolutional layer stack as the classification head. This configuration resulted in 6.4M parameters, 1.7B FLOPs, and 1.4,ms latency, achieving a classification accuracy of 86.2%.

**Table 5:** Ablation study on structural components of CNATNet.

Model	C2S2	AnC2f	DWClassify	FLOPs (B)	Params (M)	Acc (%)	Latency (ms)
M1 (Baseline)	–	–	–	1.7	6.4	86.2	1.4
M2 (+C2S2)	✓	–	–	1.4	6.1	86.5	1.1
M3 (+AnC2f)	–	✓	–	5.1	10.5	95.4	2.3
M4 (+DWClassify)	–	–	✓	1.5	6.0	86.1	1.0
M6 (+C2S2+AnC2f)	✓	✓	–	4.9	10.1	95.5	2.5
M7 (+C2S2+DWClassify)	✓	–	✓	1.2	5.8	86.1	0.8
M8 (+AnC2f+DWClassify)	–	✓	✓	5.0	10.1	95.1	2.4
M9 (CNATNet)	✓	✓	✓	4.6	9.8	95.6	1.9

Model M2 introduced the lightweight C2S2 backbone, which reduced FLOPs from 1.7B to 1.4B and parameters from 6.4M to 6.1M, with latency reduced to 1.1,ms. Accuracy slightly increased to 86.5%, showing improved efficiency but limited gains in representational power.

Model M3 integrated the AnC2f attention-enhanced fusion module. This markedly improved accuracy to 95.4%, demonstrating its effectiveness in capturing multiscale structural information. However, the added attention increased parameters to 10.5M, FLOPs to 5.1B, and latency to 2.3,ms.

Model M4 replaced the dense classifier with the proposed DWClassify head. This lightweight adjustment reduced parameters to 6.0M and FLOPs to 1.5B, achieving the lowest latency of 1.0,ms. Accuracy was comparable to the baseline (86.1%), highlighting DWClassify’s role in efficiency rather than accuracy enhancement.

When combining modules, Model M6 (C2S2+AnC2f) achieved 95.5% accuracy with 10.1M parameters and 4.9B FLOPs, showing that C2S2 complements AnC2f by reducing part of its computational burden. Model M7 (C2S2+DWClassify) provided the most efficient setting, with only 5.8M parameters, 1.2B FLOPs, and 0.8,ms latency, though accuracy remained at 86.1%. Model M8 (AnC2f+DWClassify) yielded 95.1% accuracy with 10.1M parameters, 5.0B FLOPs, and 2.4,ms latency, achieving a balance between accuracy and efficiency compared to using AnC2f alone.

Finally, the complete CNATNet (M9), which integrates all three modules, demonstrated the best trade-off between accuracy and efficiency. It achieved 95.6% accuracy with 9.8M parameters, 4.6B FLOPs, and 1.9,ms latency. Compared with M3 (highest accuracy but heavy) and M7 (highest efficiency but low accuracy), CNATNet effectively balances both aspects, confirming the synergistic contribution of C2S2, AnC2f, and DWClassify.

Overall, the ablation results confirm that each proposed module contributes uniquely: C2S2 improves backbone efficiency, AnC2f significantly enhances feature fusion and accuracy, and DWClassify ensures lightweight classification. Their joint integration enables CNATNet to achieve superior performance while maintaining low computational overhead, meeting the requirements of automated safflower filament classification tasks.

### 3.4 Embedded deployment and system implementation on Jetson Orin Nano

**Figure 11:**
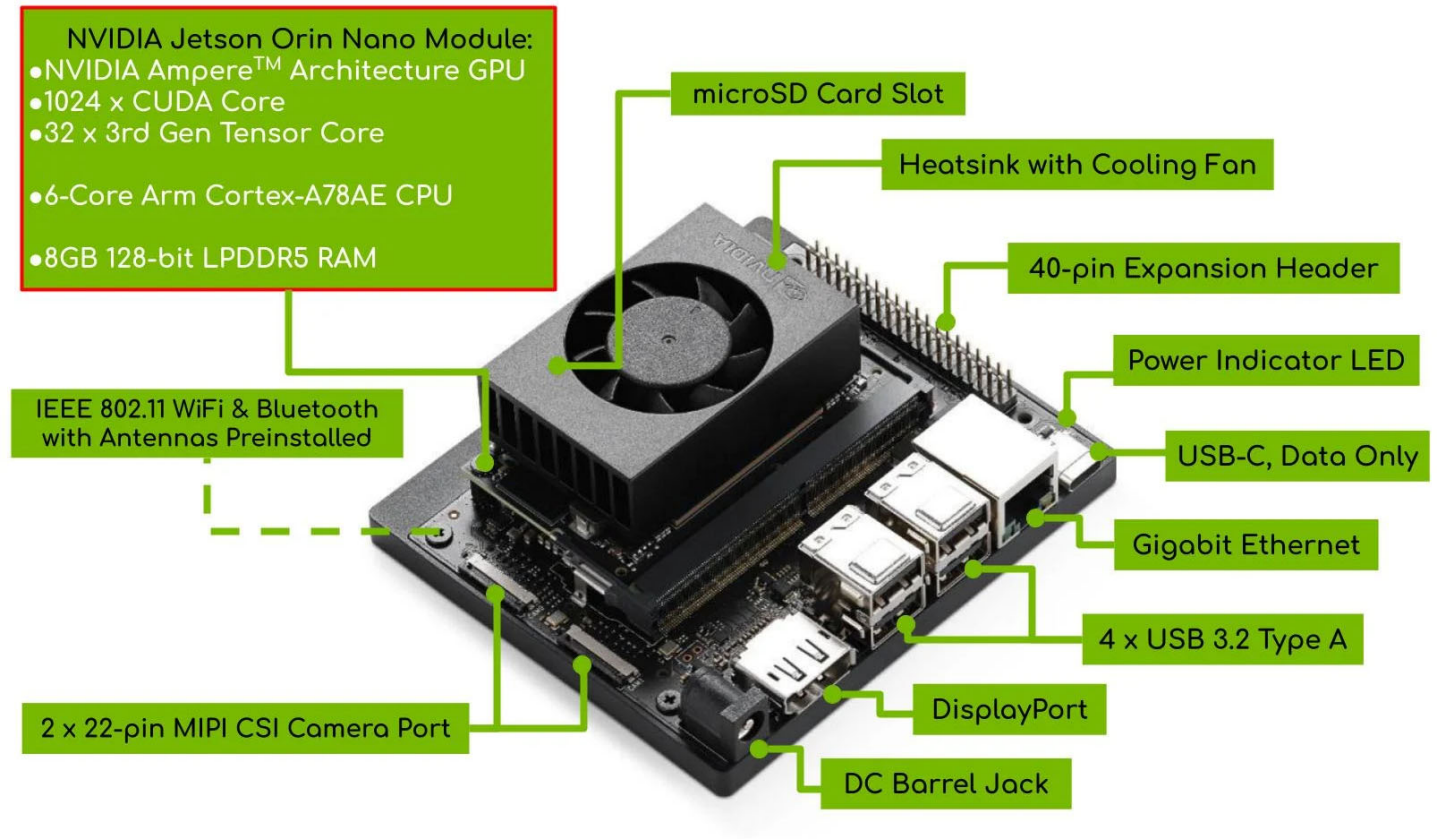
Jetson Orin Nano physical image.

With the increasing computational capabilities of modern embedded AI hardware, edge devices have become viable platforms for deploying deep learning models in real-world agricultural applications. To evaluate the practicality of the proposed model in resource-constrained scenarios, we deployed our lightweight classification network, CNATNet, on the Jetson Orin Nano platform. This deployment demonstrates the model’s suitability for real-time, on-device safflower filament grading without reliance on high-end GPU servers. The Jetson Orin Nano, based on the ARM architecture and optimized for AI inference tasks, provides a compelling balance between energy efficiency and computational performance. A visual overview of the deployed system is presented in **Figure 11**, while detailed hardware specifications are summarized in **Table 6**.

**Table 6:** Key specifications of Jetson Orin Nano (8GB).

Name	Jetson Orin Nano (8GB)
CPU	6-core ARM Cortex-A78AE @ 1.7GHz
GPU	32-core NVIDIA Ampere, 67 TOPS AI compute
Memory	8GB LPDDR5, 102.4GB/s
Storage	128GB external NVMe SSD
Network	1 × Gigabit Ethernet
USB	4 × USB 3.2 Gen2

#### 3.4.1 Model deployment

To support intelligent plant recognition in embedded agricultural scenarios, this study adopts a coarse-to-fine classification framework. Specifically, a cluster-level (coarse) classification is first performed to quickly filter and group safflower samples, followed by a fine-grained filament-level recognition to achieve precise grading. The proposed CNATNet model, which integrates lightweight convolutional and attention mechanisms, was initially trained and optimized on a high-performance local workstation. The final optimized version was then deployed to the Jetson Orin Nano platform for real-time on-device inference. This deployment not only verifies the model’s efficiency and robustness under low-power constraints, but also highlights the advantage of its lightweight design, which enables stable inference on embedded hardware at 63.29 FPS with only 15 W power consumption. These results demonstrate the potential of CNATNet in enabling intelligent, embedded plant classification for modern agricultural systems.

#### 3.4.2 Test results and analysis

**Figure 12:**
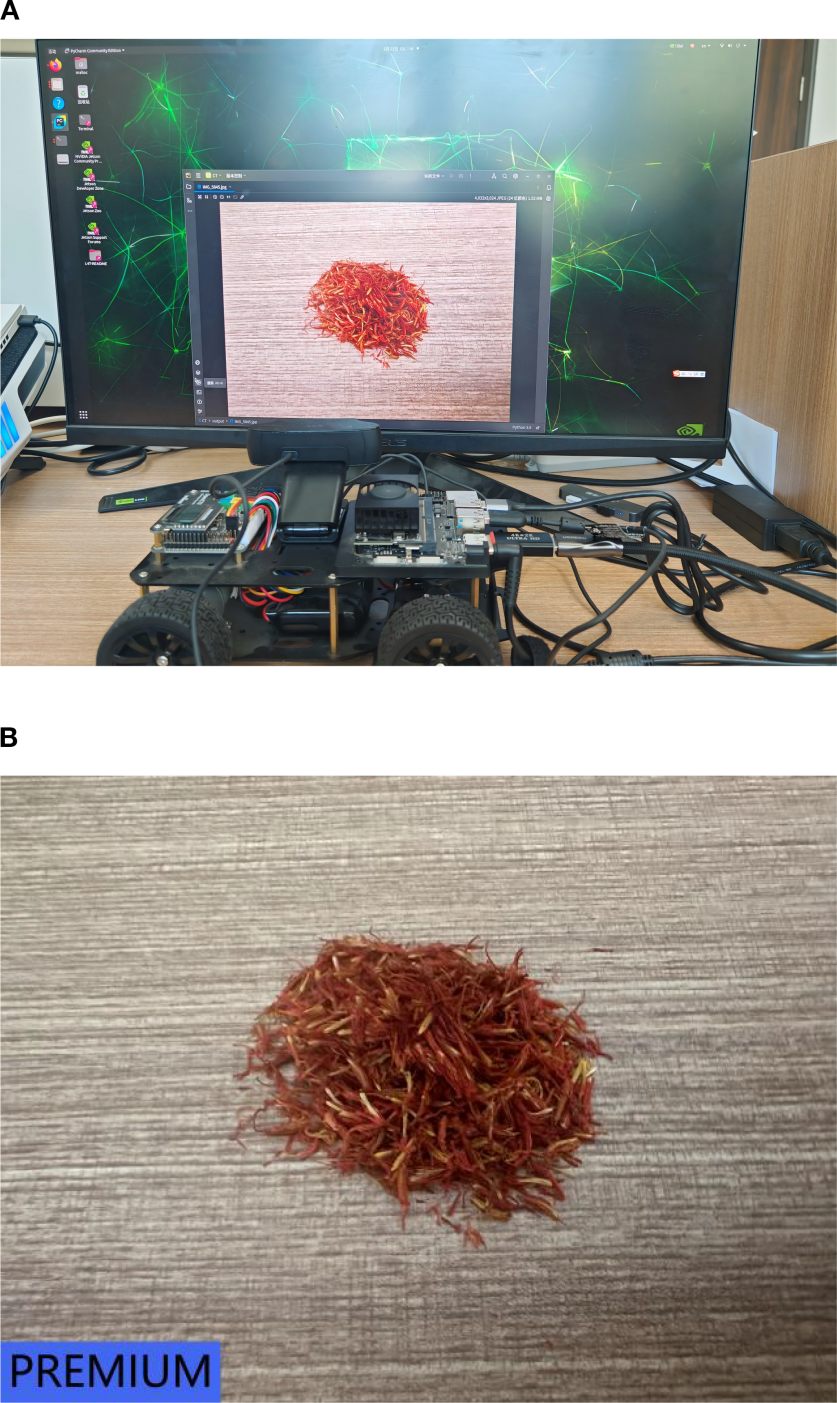
Visual demonstration of real-time safflower classification using CNATNet deployed on Jetson Orin Nano. **(A)** On-device inference setup. **(B)** Prediction result: *PREMIUM* grade.

In order to evaluate the real-time performance of the CNATNet-based safflower classification system on the Jetson Orin Nano platform, a live camera-based testing method is employed, enabling real-time recognition of safflower clusters and filaments under practical deployment conditions. The overall process is illustrated in **Figure 12**, where the camera captures input images, which are then processed by the deployed model for on-device inference. The classification results are displayed in real time, demonstrating the effectiveness of the system in practical scenarios. Specifically, subfigure (A) presents the actual on-device deployment setup, including the Jetson Orin Nano, camera, and display screen, while subfigure (B) shows the corresponding real-time prediction results with the classified safflower grade, confirming that the lightweight CNATNet model achieves both accuracy and efficiency in embedded agricultural environments.

## 4 Conclusion

In this study, a lightweight hybrid network, CNATNet, was proposed for safflower filament classification. The architecture integrates multi-branch convolutional feature extraction, attention-enhanced fusion, and a lightweight classification head, achieving a balance between accuracy and computational efficiency.

Experimental evaluations showed that CNATNet achieved 95.6% classification accuracy, with markedly reduced parameters, floatingpoint operations, and inference latency. These results confirm that the proposed lightweight design meets the practical requirements of real-time deployment in resource-constrained agricultural environments. Furthermore, deployment on the Jetson Orin Nano platform demonstrated stable real-time performance at low power, validating its suitability for embedded agricultural grading tasks. The lightweight design principles adopted in CNATNet provide a feasible solution for fine-grained quality assessment, with potential applications extending beyond safflower classification to other agricultural and industrial scenarios.

Nevertheless, challenges remain under complex environmental conditions such as variable illumination, occlusion, and background interference, which may affect robustness. Future work will focus on improving generalization through adaptive illumination normalization, domain-specific data augmentation, and lightweight multimodal fusion strategies.

## Data Availability

The original contributions presented in the study are included in the article/supplementary material. Further inquiries can be directed to the corresponding author.
